# Ten Years of ‘Flying the Flag’: An Overview and Retrospective Consideration of the Active School Flag Physical Activity Initiative for Children—Design, Development & Evaluation

**DOI:** 10.3390/children7120300

**Published:** 2020-12-16

**Authors:** Sarahjane Belton, Úna Britton, Elaine Murtagh, Sarah Meegan, Christina Duff, Jamie McGann

**Affiliations:** 1 School of Health & Human Performance, Dublin City University, D09 V209 Dublin, Ireland; una.britton2@mail.dcu.ie (Ú.B.); sarah.meegan@dcu.ie (S.M.); christina.duff4@mail.dcu.ie (C.D.); Jamie.mcgann@dcu.ie (J.M.); 2 Department of Physical Education & Sport Sciences, University of Limerick, V94 T9PX Limerick, Ireland; elaine.murtagh@ul.ie; 3 Physical Activity for Health Research Cluster, Health Research Institute, University of Limerick, V94 T9PX Limerick, Ireland

**Keywords:** physical activity, active schools, health promotion, primary education

## Abstract

Whole-school physical activity (PA) promotion programmes are recommended to increase youth PA. Evaluation of programmes is essential to ensure practice is guided by evidence. This paper evaluates the Active School Flag (ASF), a whole-school PA promotion programme in Ireland, using the Reach, Effectiveness, Adoption, Implementation, and Maintenance (RE-AIM) framework. ASF was evaluated across three levels—(1) administration, (2) application, (3) outcomes—using a mixed-methods case study design. Existing data sources were reviewed, the programme coordinator was interviewed, and a pilot study was conducted to investigate impact on 3rd and 5th class students (3 schools, *n* = 126 students, age range 8–12 years). In-school Moderate to Vigorous Physical Activity (MVPA; by accelerometery), motivation for PA (BREQ), PA self-efficacy (PASES), school affect and peer social support (Kidscreen27) were measured pre-programme (0 months), post-programme (8 months), and at retention (12 months). Teacher perceptions of classroom behaviour (CBAST) were also measured pre- and post-programme. ASF has been successful in engaging 46% of primary schools nationally. Students’ in-school moderate–vigorous PA increased in all pilot-study schools from pre-programme to retention (η^2^ = 0.68–0.84). ASF programme design facilitates implementation fidelity, adoption and maintenance through buy in from schools and government stakeholders. ASF presents as an effective PA promotion programme in the short-to-medium term for primary schools. This RE-AIM evaluation provides evidence of ASF effectiveness, alongside valuable findings that could support programme improvement, and inform future similar programmes.

## 1. Introduction

Physical activity (PA) is associated with numerous positive health outcomes in youth [1,2], with individuals who are active as children more likely to sustain their PA levels into adolescence [3,4]. Current national [5] and international [6] guidelines recommend that children and youth should engage in at least 60 min of moderate–vigorous physical activity (MVPA) each day. The majority of youth worldwide, however, do not meet these guidelines, with recently published data indicating that, globally, only 22% of males and 15% of females aged 11–17 are sufficiently active [7]. In Ireland, 17% of primary school children (23% males and 13% females) report engaging in 60 min of MVPA daily [8]. This physical inactivity ‘crisis’ means that children are at increased risk of developing non-communicable diseases such as heart disease and diabetes in later life [6,9].

Recognition of the significant health and economic costs associated with physical inactivity has led to a focus on PA promotion strategies at both the national and international policy levels [10,11]. Strengthening national implementation of whole-school PA promotion programmes is proposed as a way to tackle the existing low levels of PA [10,11], particularly in light of children spending almost half their day in the school environment [12]. In the USA, the Comprehensive School Physical Activity Program (CSPAP), a whole-school initiative which highlights the importance of collaboration between relevant stakeholders to effect behaviour change, has been used nationally to promote PA in childhood [13]. Recently, both the UK (Creating Active Schools Framework: CAS) and Australia (Physical Literacy Framework) have published PA promotion frameworks which similarly recognise the essential need for whole-school approaches, and buy in from multiple stakeholders in these efforts [14,15]. Both CSPAP and CAS are grounded in the social-ecological framework, while the Australian Physical Literacy Framework is built around the four components of physical literacy—motivation, confidence, competence, and knowledge and understanding [16].

Irrespective of specific paradigms used by individual PA promotion initiatives, it is recognised that to successfully increase PA engagement, initiatives must be multicomponent (for example this could include targeting the individual child’s behaviour at school, as well as perhaps their school environment, and their parents/teachers), garner support from a range of relevant stakeholders, and encompass multiple factors known to support positive PA behaviours [17]. Lessons learned from existing whole-school PA promotion programmes suggest that there are a number of key ingredients required to support effective PA behaviour change both in school, and beyond the school setting [14,18]. Physical education (PE) is often the pillar from which PA promotion in schools is led. Quality PE has the potential to foster actual and perceived movement competence and confidence in children, positively impacting motivation towards long-term PA engagement [19]. It is acknowledged, however, that PE alone cannot solve the problem of low PA levels either within or outside of school, and that a multicomponent whole-school approach to PA promotion (which may well include PE, but not be limited to this) is needed to support lasting change [18]. To that end, children must be provided with additional opportunities to experience fun co-curricular PA, using a multistakeholder approach whereby school, local community, and wider stakeholders (e.g., public health and physical activity specialists) all contribute to establishment of positive activity habits [14,18]. The importance of school–community partnerships is highlighted to enable knowledge exchange (e.g., provision of information relating to how, why and when children can be active in the school community) where the goal of PA promotion is to increase PA not just during school time, but beyond the school day as well [18].

Evidence from existing programmes has highlighted several challenges when implementing whole-school PA initiatives. A recent review highlighted the potential for implementation infidelity, with interventions being well positioned in many cases to affect change, but components not implemented the way they were intended, resulting in a negation of the desired effects of school-based PA promotion programmes [20]. Indeed in many cases, it is a lack of detail or information on intervention fidelity that can be the challenge [20]. Multicomponent school-based PA initiatives in particular have more ‘parts’ and can take time to embed and see results, and as such can be perceived by educators as more challenging to implement with fidelity [21]. Thus, access to training, and continual professional programme support structures, should be available to programme coordinators to ensure implementation fidelity can be sustained [18,22]. In a similar vein, however, whole-school PA promotion programmes intended for widespread use should be flexible and allow for individual variation to account for differences in social and physical environments [18,21,22].

Where the goal is to link evidence with practice, it is imperative to document the method by which successful real-world PA initiatives are implemented [23]. As such, publication of theoretical frameworks for PA promotion is important to facilitate our understanding of the development and implementation of successful programmes. Ireland’s Active School Flag (ASF) programme is a whole-school, multicomponent PA promotion initiative, developed by the Department of Education and Skills and supported by the Department of Health under the ‘Healthy Ireland’ programme (www.activeschoolflag.ie). The goal of the Active School Flag is to enable ‘more children to be more active, more often’. ASF takes an ecological approach (Bronfenbrenner, 1979) and was developed as a series of pragmatic steps and decisions made by practitioners in the field to provide an intervention that could help address low levels of PA in Irish schools. It grew organically, with the impetus for such a programme coming on the back of growing evidence supporting that low levels of physical activity were an issue [24,25,26]. When the components of the intervention are considered, along with the framework that supports its implementation (both of which are presented later in the paper), it is clear that while not the foundation from which it evolved, it maps clearly with several principles and frameworks which are now accepted as best practice in school based intervention work (including the previously mentioned CSPAP [13], CAS [14], and the Australian Physical Literacy Framework [15]). Since its inception in 2009 the ASF has been adopted by more than 2000 primary schools in Ireland. For the past ten years, the ASF have been ‘flying the flag’ for physical activity in Irish school communities, continuously refining and developing programme content based on both research findings and teacher feedback [21]. Considering the adoption success of the ASF in Irish primary schools, and its continued support at school and government department level, an evaluation of the structure and outcomes of the ASF offers the opportunity to more broadly evaluate the potential of this whole-school PA promotion initiative in a real-world setting. Research on the ASF indicates that schools are receptive to this programme and intrinsically motivated to engage with the ASF process for a number of reasons, including their belief that PA is important for tackling levels of obesity in Ireland, and recognition of how low levels of motor competence can negatively impact PA participation [21]. In addition, the very idea of having a ‘physical flag’ to display at the school gates as a sign of status and recognition has been highlighted as a key ‘extrinsic’ motivation for schools to engage with the programme [21,27]. The potential for the ASF to have a lasting impact on the school environment has also been highlighted, with studies reporting lasting changes on schools’ awareness of the importance of PA, on PA equipment and facilities, and on opportunities to engage in PA [21,28]. Barriers to engagement in the ASF have also been highlighted, with schools identifying time commitments and paperwork as potential disincentives [21,28]. In addition, findings suggest that socially disadvantaged schools may find it more difficult to implement some aspects of the ASF compared to socially advantaged schools [21].

Published research findings, along with teacher experiences and feedback, have to date been used to inform incremental changes to the ASF implementation process over the past ten years. Now, after ten years of implementation, there presents an opportunity to evaluate the ASF in a broad context, with a view to sharing lessons learned from this national PA promotion initiative with others who are looking to develop or adopt similar whole-school PA promotion programmes. To that end, this study aims to broadly evaluate the ASF programme using the Reach, Effectiveness, Adoption, Implementation, and Maintenance (RE-AIM) framework [29]. The RE-AIM framework has been used previously to evaluate school-based PA interventions [30,31,32], the primary goal of such evaluation being to enable successful interventions to be applied to a range of different settings and populations, maximising the positive impact on public health of these proven successful interventions [29]. RE-AIM was chosen as a framework on which to align the current evaluation as it allows for interrogation of ASF across five domains highly relevant to informing practical decisions and improvements that may be made to the intervention going forward.

## 2. Materials and Methods

The current paper is concerned with a RE-AIM evaluation of the ASF across three levels: (1) administration, (2) application and (3) outcomes. At the administration level, the overarching organisation and function of the ASF in terms of programme (R)each, (A)doption, (I)mplementation fidelity, and (M)aintenance was evaluated. The application level was concerned with schools’ (I)mplementation fidelity of the ASF, while the outcomes level was concerned with the (E)ffectiveness of the ASF on outcomes such as MVPA, motivation for PA, PA self-efficacy, school affect, and behaviour. A summary of the definitions and data sources used for each RE-AIM dimension are provided in Table 1. This study involved a review of existing data sources (e.g., literature and research articles on the ASF development, implementation and effectiveness to date), an interview with the ASF programme coordinator and a pilot evaluation of the impact that ASF participation has on children from three schools across a period of 12 months. Further details of this mixed-methods case study approach are outlined below. Ethical approval was provided by the institutional Research Ethics Committee (reference number: DCUREC2018_168).

### 2.1. Intervention—ASF Programme Overview

The ASF aims to get ‘more schools more active more often’ (www.activeschoolflag.ie). The ASF identifies four ‘pillars’ which are supportive of PA promotion in schools, and which schools are required to address in order to achieve the flag. The four pillars are: (1) physical activity, (2) physical education, (3) community partnerships (pupils, parents, local communities and national agencies) and (4) Active School Week (www.activeschoolflag.ie). Schools are first required to self-evaluate their current provision under three pillars (the Active School Week pillar is not included) guided by questions set out by the ASF. Questions are designed for discussion and agreement at whole staff level. Completing the questionnaire helps schools to identify areas of strength and areas that require further attention. These areas can then be tackled using ASF ‘Success criteria’. Ultimately, the ASF is awarded to schools who achieve all ‘success criteria’ set out by the ASF across each of the four pillars.

Schools must be able to confirm that they are meeting all of the ASF criteria (Table 2) for each of the pillars and provide visual evidence to that effect on their school website or via a PowerPoint before they are awarded the ASF and ASF status. The ASF website and ASF social media provides examples of how schools have implemented these success criteria in different ways depending on their social and physical environment (www.activeschoolflag.ie). Schools complete an online application form, marking off the success criteria as and when they are achieved. Once completed, a screening application is sent by the school to the ASF Screening Committee who review the application and recommend the school for an accreditation visit or, recommend areas that need to be addressed before an accreditor will visit. If a school is recommended for an accreditation visit, the ASF accreditor visits the school to verify the school’s application, acknowledge the efforts of all those that contributed to the ASF process, and award the flag if the school can demonstrate it is meeting the success criteria. Once awarded, a school retains its ASF for a period of three years, after which it must apply to ‘renew’ its status.

### 2.2. Participant Recruitment

#### 2.2.1. Administration Level: ASF National Coordinator

The ASF National Coordinator, who has directed the ASF for the past 10 years, was invited via email to participate in a semi-structured interview to discuss the setup, structure and evolvement of the ASF over the course of the last 10 years.

#### 2.2.2. Application Level: Schools

Schools were recruited by the research team at ASF workshops at the beginning of the 2018–2019 academic year. At these workshops, a researcher provided attendees with information about the research study. To be eligible for inclusion schools had to commence the ASF programme for the first time that academic year (2018–2019), had to be co-educational and had to be designated disadvantaged (DEIS) by the Department of Education and Skills. DEIS schools are those where students are at risk of educational disadvantage due to their social or economic position [33]. Research in an Irish context shows that there is a socioeconomic divide in sport participation [8] and health-related physical fitness levels [34] among Irish children, to the detriment of those from lower socioeconomic communities. There are currently 692 designated disadvantaged primary schools in Ireland, out of a total of 3107 primary schools [35]. After the ASF workshop, attendees from nine eligible schools provided contact details and were sent an email inviting their school to participate in the research study, with details of what participation would involve. Three co-educational schools who were about to begin implementation of the ASF programme replied expressing interest in participating in the research and were recruited to this study.

#### 2.2.3. Outcomes Level: Students and Teachers

Participants were students and teachers of the recruited primary schools. Students who were in 3rd (children aged 8–10 years) and 5th class (children aged 10–12 years) in September 2018 were invited to take part in this study. A high number of children consented to participate with 126 children out of possible 172 across all three schools providing data across the research period. All class teachers within the recruited primary schools were invited to participate by the teacher in charge of ASF coordination in that school, with 39 teachers across the three schools participating. It is worth noting for context, that in Irish primary schools there are no specialist PE teachers, generalist teachers are responsible for teaching PE. PE is a recognised subject on the curriculum which schools are expected to deliver. Given the nature of PE and its role in PA, it is one of the pillars of the ASF intervention. Participants were provided with a plain language statement detailing the purpose of this study and what participation would entail. Participant assent, and parental consent for juvenile participants, was obtained prior to data collection.

### 2.3. Measurement

#### 2.3.1. Administrative Level

Three sources of data were used for analysis of the ASF at the administration level. These were: (1) a semi-structured interview with the ASF National Coordinator, (2) data from the ASF website (www.activeschoolflag.ie), and (3) data from the ASF Administration Office collected via email correspondence. The interview schedule for the ASF National Coordinator included 15 questions focusing on the development of the ASF from its inception ten years previously, to its current structure. Questions were framed within the context of the five RE-AIM components [29]. Due to this part of the research being carried out during the Covid-19 pandemic, the interview was conducted using Zoom (Zoom Video Communications, Inc., San Jose, CA, USA). Kite and Phongsavan (2017) [36] have reported that the quality of data collected through online communication platforms like Zoom is similar to that which is collected in face-to-face discussions. The ASF website (www.activeschoolflag.ie) was searched for information on current application, engagement, and awarding processes of the ASF, while the ASF Administration Office was contacted via email for data relating to adoption of the ASF programme by primary schools.

#### 2.3.2. Application Level

Implementation of the ASF by each school was evaluated using the schools’ success criteria document (Table 2). This document was completed by each school as part of the ASF process, and subsequently shared with the research team. The document details which of the ASF’s success criteria were achieved during the process.

#### 2.3.3. Outcomes Level

For the purpose of student outcomes, the ASF was considered an intervention programme which the students were being exposed to. Student outcomes (in-school MVPA, school affect, peer social support, motivation to be physically active, and PA self-efficacy) were measured at 0 months (pre-intervention, September 2018), 8 months (post- intervention, May 2019), and 12 months (retention, September 2019).

Student in-school PA levels were measured using Actigraph (Actigraph LLC) accelerometers (models GT1M and GT3X; Pensacola, FL, USA)), which were set to capture data in 10 s epochs (Esliger et al. 2005). Student participants wore the accelerometer above the iliac crest of their right hip, during school hours for a period of five days. Teachers were instructed by the research team to remind student participants to put the accelerometer on arrival at school and to remove the accelerometer (and leave on the school desk) when leaving at the end of the school day.

School affect and peer social support were measured using the School Environment and Social Support and Peers subsections of the Kidscreen27 Questionnaire [37]. Both dimensions contain four items, answered on a five-point Likert scale. Shannon et al. (2017) have reported acceptable internal reliability for the School Environment (Cronbach’s α = 0.72) and Social Support and Peers (Cronbach’s α = 0.72) dimensions of the Kidscreen27 in a sample of Irish children of low socioeconomic status [38].

Motivation to be physically active was measured using the Behavioural Regulation in Exercise Questionnaire—Altered (BREQ) [39]. The BREQ-Altered contains 12 items, answered on a five-point Likert scale ranging from ‘not true for me’ to ‘very true for me’, with items pertaining to four types of motivation (intrinsic, identified, introjected, and external motivation) included. The BREQ has been deemed to have satisfactory reliability (Cronbach’s α = 0.59–0.77 for subscales) and validity (confirmatory factor analysis > 0.9; RMSEA < 0.05) [39]. PA self-efficacy was measured using the Physical Activity Self-Efficacy Scale (PASES) [40]. Confirmatory factor analysis indicates acceptable validity of this tool for measuring PA self-efficacy in children (comparative fit index (CFI) = 0.93–0.95; root mean square error of approximation (RMSEA) < 0.05) [40].

Teacher perceptions of student behaviour were measured at two timepoints: 0 months (September 2018) and 8 months (May 2019), using the Classroom Behaviour and Asset Survey: Teachers (CBAST). The CBAST is designed to provide a snapshot of a teacher’s perception of problem behaviours, and asset behaviours, of an entire class of students [41]. The CBAST has good internal consistency (α = 0.97) for asset behaviours and for problem behaviours (α = 0.95) [42]. The adapted version of the CBAST was used in the current study, which includes 10 of the 60 items in the long form, reflecting four asset behaviours (items 1–4), and six problem behaviour items (items 5–10). Previous studies have indicated an association between these ten items and children’s PA participation [43,44]. Item response options were scaled from 0 to 7 indicating the proportion of the class engaging in specific behaviours (0 = 0 students, 1 = 1-2 students, 2 = a few students, 3 = approximately ¼ of the class, 4 = approximately ½ of the class, 5 = approximately ¾ of the class, 6 = most of the class, 7 = all of the class) [45].

### 2.4. Data Processing and Analysis

The ASF National Coordinator’s interview was recorded using Zoom (Zoom Video Communications, Inc., San Jose, CA, USA) and transcribed verbatim. The second and last authors coded the interview by individually reading through the transcript and highlighting text segments to be coded under each of the five RE-AIM categories. A back and forth process between authors ensued until agreement on final coding was reached. Each school’s ASF success criteria document (Table 2) was analysed to identify which of the ASF success criteria were achieved and which were not. Accelerometer data were processed in Actilife (version 6.13.3). Mean minutes of in-school MVPA were calculated for each student. The first and last days of wear were excluded to account for subject reactivity [46]. Student participants who wore the accelerometer for a minimum of five hours on three school days were deemed as having met the wear criteria [47] and were included in the analysis. MVPA was calculated using the Actigraph software, applying the Evenson et al. (2008) cut-points [48]. Periods of greater than 20 min of consecutive zero counts in the accelerometer data were defined as non-wear time and discounted from the data [49,50].

All raw quantitative data were entered and analysed in SPSS27. Mean scores ranging from 0 to 5 were calculated for each participant for school affect and peer social support from the raw scores of all items in the School Environment and Social Support and Peers dimensions of the Kidscreen27. Specific items were reverse scored so that higher numbers represented more positive wellbeing. Raw scores for each of the 12 items on the BREQ-Altered were entered into SPSS. Responses to the external and introjected motivation items were reverse scored so that higher scores across all items represented higher levels of positive motivation behaviours. Scores on each of the 12 items were then summed to create a total motivation score. Raw scores on each item of the PASES [40] were summed to create a total score for PA self-efficacy.

Two subscales, one for asset behaviour and one for problem behaviour, were created from the CBAST data by averaging the items pertaining to each scale [51].

One-way repeated-measures ANOVA were used to analyse changes in in-school MVPA, school affect, peer social support, motivation for PA, and self-efficacy for PA within schools over three timepoints. Eta-squared (η^2^) was used to estimate effect size estimates of these changes over time and were interpreted as 0.01 = small, 0.06 = medium, and 0.14 = large [52]. Mixed between-within ANOVA were used to analyse differences in the pattern of change in outcome variables over time between schools, and between class groups within each school. Paired samples t-tests were used to analyse changes in asset and problem behaviour over two timepoints. Effect size estimates of mean differences in behaviour between time 1 and time 2 were calculated. Hedges *g*_av_, to account for the small sample sizes, and the common language effect size (CL ES) were reported, following the recommendations of Lakens (2013). The CL ES converts the effect size into a percentage, making it intuitively easier to interpret [53]. It expresses the probability that an individual’s score at one timepoint will be different to their score at the next timepoint [53].

## 3. Results

Definitions of, and data sources for, each RE-AIM dimension at each level of analysis are summarised in Table 1.

### 3.1. Administrative Level

#### 3.1.1. Reach

**Figure 1:**
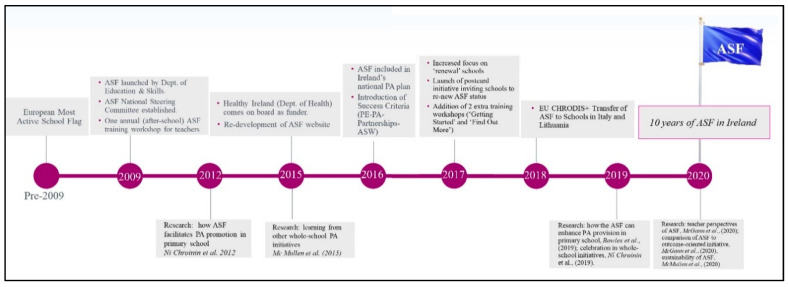
Active School Flag timeline.

**Figure 2:**
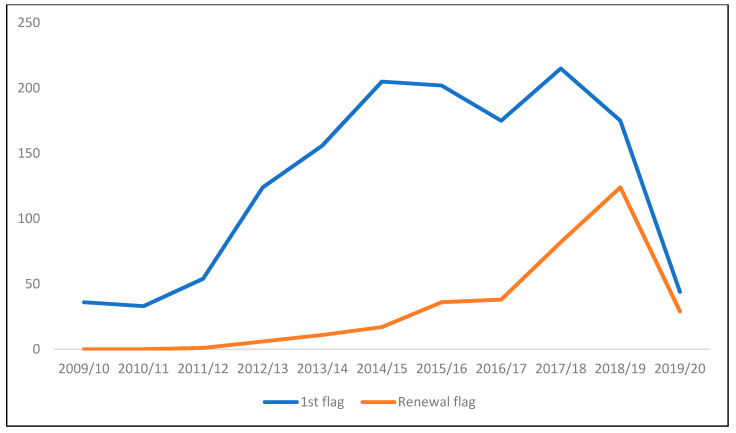
Number of schools receiving their first flag and number of schools renewing their flag each academic year. Note: Covid-19-related school closures from March to June 2020 likely impacted applications and renewals for the ASF programme.

Being a whole-school initiative, the ASF’s goal is to reach every pupil in every school in which it is implemented. At a national policy level, the ASF is part of Ireland’s National Physical Activity Plan [11], developed by Healthy Ireland under the auspices of the Department of Health 2016 (www.activeschoolflag.ie). A timeline showing the development of the ASF is shown in Figure 1. The support of the ASF by Healthy Ireland since 2015 has brought additional funding to expand the reach of the programme. Figure 2 shows this growth in school engagement over time.

ASF National Coordinator: *‘Being part of the National Physical Activity Plan, brings in additional funding from Healthy Ireland to run the initiative. One of the most significant benefits of increased funding was that it facilitated increased training opportunities (i.e., our ‘Getting Started’ and ‘Nearly There’ workshops) and the provision of resources for schools to help them implement the success criteria in an exciting way.’*

Additional funding also facilitated the recruitment of a part-time administrator and a part-time development officer at primary and post-primary levels. It enabled the role of the ASF National Coordinator to move from a part-time to a full-time position and maintain the central ASF office that keeps constant communication with stakeholders. Funding was also used to support reach through design of ASF promotional videos, design and maintenance of ASF website, provision of increased training opportunities (‘Getting Started’ and ‘Nearly There’ workshops at Education Centres) for teachers/coordinators, development and maintenance of an ASF ‘online application’ portal as well as ASF social media accounts that have proved increasingly popular. Funding supported an opportunity to develop strong branding and the translation of ASF goals into attractive booklets, leaflets and literature that has been shared with thousands of teachers both online and ‘on the ground’ at training opportunities.

To increase the reach of the programme, adaptations have also been made since the programme was initiated ten years ago. Feedback and communication between the ASF team and teachers was highlighted by the ASF National Coordinator as an imperative part of successfully evolving the ASF to reach more schools. In addition, the coordinator added the importance of utilising research findings as inputs to improve the process going further.

ASF National Coordinator: *‘It’s a two-way process….one of the strengths of ASF is that we are good at listening. We take on board every piece of feedback sent through to us from schools and that shapes the process. Equally we pay attention to what the research says, the positive and the negative, and we are guided by that. Teachers know what will work and what won’t, what will fit into the school day and what won’t; so it makes sense to ask them…… and that shapes the process.’*

Additional adaptions to facilitate reach include the removal of extra-curricular activities and ‘active travel’ from the ASF process from 2016/2017 onwards, mainly because these activities took place outside of the teaching day. Primarily the ASF wanted schools to focus their efforts on the teaching day when all pupils were with their teacher, ensuring that all ASF activities were inclusive. They also felt that the process should not require teachers to work outside of their teaching hours.

ASF National Coordinator: *‘we couldn’t build in a requirement to the process that teachers had to stay back after school.’*

#### 3.1.2. Adoption

**Figure 3:**
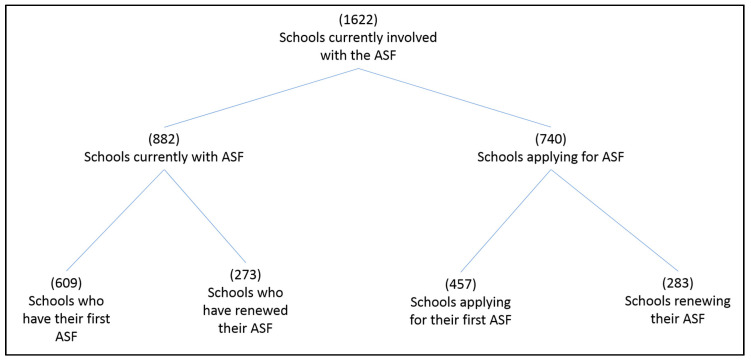
Breakdown of schools currently engaged with the ASF.

Overall, since its inception 10 years ago, 2635 primary schools have registered with the ASF (Figure 3). This means that almost 85% of Irish primary schools have, at some point, registered with the ASF initiative, thus indicating their interest in learning more about the initiative. In total, 1419 primary schools have attained at least one flag (46% of all primary schools), with 314 of these schools renewing their flag at least once.

There are currently 1622 schools involved with the ASF, either in possession of a flag, or in the application process (Figure 3).

Of the 882 schools who currently have the ASF, 147 are designated by the Department of Education and Skills as disadvantaged schools [35]. This indicates that approximately 21% of designated disadvantaged schools compared to 32% of non-designated disadvantaged schools [35] are in possession of an ASF. There are no statistics available at this time for a breakdown of designated disadvantaged schools among those in the application process.

#### 3.1.3. Implementation

The ASF initiative is designed to maximise implementation fidelity, in terms of how schools implement the programme, and how the ASF is awarded. Changes to improve the implementation fidelity of the ASF have been made throughout the past ten years. One significant change outlined by the ASF National Coordinator was the introduction of ‘success criteria’ in the form of a checklist used by schools to guide and document their implementation of the ASF programme (Table 2). Recently, this checklist has been provided as an online form, which allows schools to track their own progress and then apply for accreditation when they have said ‘Yes’ to all success criteria. Additional workshops (‘Getting Started’ and ‘Nearly There’) were introduced at the same time as the refined success criteria documents, enabling the ASF to offer practical advice to schools/coordinators in terms of how they could achieve the criteria. Workshops also intended to support a community of practice, with teachers encouraged to share ideas and their experiences.

ASF National Coordinator: *‘Success criteria were only introduced around five or six years ago, based on feedback from the schools. They wanted success criteria for the Active School Flag. They [the success criteria] came about from teacher feedback, and in an effort to get rid of the heavy paperwork.’*

Implementation fidelity in terms of how schools are assessed and awarded the ASF is also a key concern. Within the ASF there are trained teams of screeners and accreditors. Screeners and accreditors attend a training day at the beginning of the school year, and new screeners and accreditors are required to do a ‘shadow visit’ where they visit a school with an experienced member of the ASF team to learn about the process. When a school is satisfied that they have addressed the success criteria they submit an online application which is then forwarded to the ASF Screening Committee for review. Once the Screening Committee is satisfied, an ASF accreditor visits the school to verify the school’s application, and to award the flag if the school can demonstrate it is meeting the success criteria. The importance placed on continuous communication and training for screeners and accreditors within the ASF process, as well as obtaining high quality individuals for these roles, was highlighted by the ASF National Coordinator.

ASF National Coordinator: *‘There’s an accreditation training day in September every year. Before you do your first accreditation visit, you do a shadow visit with somebody else, an experienced accreditor brings you out.’…*

‘Accreditors are recommended through the Education Centre….they have a teaching background, are familiar with Irish schools, they ‘get’ schools’…

‘There’s constant communication with the team, screeners, accreditors, they all work together to clarify things.’

#### 3.1.4. Maintenance

In 2017/2018, the ASF started to actively promote the ‘renewal’ process. This process was intended to encourage schools who had previously been awarded the ASF to demonstrate their continued commitment to be being an ASF school. The initial ASF award is valid for a three-year period after which, schools would have to complete the renewal process to retain their ASF status. Since the introduction of the renewal process, 314 schools have renewed the flag at least once. The renewal process represents a concerted effort on the part of the ASF committee to promote the maintenance of ASF implementation with high fidelity. Schools intending to officially ‘renew’ (or maintain) the ASF programme must not only demonstrate adherence to the original ASF success criteria but also complete a specific set of ‘renewal success criteria’. Once these are satisfied, a school may receive an official visit from ASF accreditors and receive their second (or third) flag. The renewal process presents as a novel way to encourage maintenance of a school-based initiative and may enhance the overall rate of renewal/maintenance.

Consistent government support for the ASF from both the Department of Education and the Department of Health, along with the inclusion of the ASF within Ireland’s National Physical Activity Plan are positive indicators and point to the ASF becoming embedded in Irish primary schools.

### 3.2. Application Level

#### Implementation

Table 2 outlines the ASF success criteria achieved by each school. Schools 1 and 2 achieved all of the success criteria across each of the four ASF pillars (Table 2). School 1 was awarded the flag in the 2019/2020 academic year, while School 2 was awarded the flag in the 2018/2019 academic year. School 3 achieved 46% of the ASF success criteria and was not awarded the flag. School 3 achieved most of their success criteria within the PA pillar (71% of the specified PA success criteria achieved), with 38% of both the PE and Partnerships success criteria completed. School 3 did not achieve any success criteria associated with the Active School Week pillar.

### 3.3. Outcome Level

Descriptive statistics for participating 3rd and 5th class students in each of the three case study schools are presented in (Table 3). In terms of outcomes, 3rd and 5th class students provided data for MVPA, motivation for PA, PA self-efficacy, school affect, and peer social support. Data on student asset and problem behaviour were provided by all class teachers, thus giving perceptions of behaviour for all students in the school (i.e., junior infants—6th class).

#### 3.3.1. Effectiveness

School 1

For School 1, results from a one-way repeated-measures ANOVA showed a significant effect for time on students’ in-school MVPA (Wilk’s lambda = 0.158, F (2, 41) = 109.07, *p* < 0.000, η^2^ = 0.842). Pairwise comparisons showed a significant increase in mean in-school MVPA from T1 to T2 and mean in-school MVPA at T3 remained significantly higher than mean in-school MVPA at T1. There was no significant interaction effect for class group and time on in-school MVPA (Wilk’s lambda = 0.991, F (2, 40) = 0.175, *p* > 0.05, η^2^ = 0.009).

There was no significant effect for time on motivation for PA (Wilk’s lambda = 0.957, F (2, 34) = 0.755, *p* > 0.05, η^2^ = 0.043), PA self-efficacy (Wilk’s lambda = 0.930, F (2, 38) = 1.423, *p* > 0.05, η^2^ = 0.070), school affect (Wilk’s lambda = 0.964, F (2, 38) = 0.699, *p* > 0.05, η^2^ = 0.036), or peer social support (Wilk’s lambda = 0.905, F (1, 39) = 4.105, *p* = 0.05, η^2^ = 0.095). Mixed between-within ANOVA’s showed no significant interaction effects for time and class group on motivation for PA (Wilk’s lambda = 0.939, F (2, 33) = 1.075, *p* > 0.05, η^2^ = 0.061), PA self-efficacy (Pillai’s trace = 0.072, F (2, 37) = 1.44, *p* > 0.05, η^2^ = 0.072), school affect (Pillai’s trace = 0.070, F (1, 38) = 2.87, *p* > 0.05, η^2^ = 0.070), or peer social support (Pillai’s trace = 0.098, F (2, 37) = 2.015, *p* > 0.05, η^2^ = 0.098).

Paired-samples *t*-tests showed a significant increase in the proportion of students displaying asset behaviour and a significant decrease in the proportion of students displaying problem behaviour from T1 to T2 for School 1. It was 97% (CL ES = 0.97) more likely that more students would display asset behaviour at T2 compared to T1, and 95% (CL ES = 0.95) more likely that less students would display problem behaviours at T2 compared to T1 (Table 4).

School 2

There was a significant effect for time on in-school MVPA for School 2 (Wilk’s lambda = 0.183, F (2, 21) = 46.84, *p* < 0.000, η^2^ = 0.817). Pairwise comparisons showed a significant increase in mean in-school MVPA from T1 to T2, and mean in-school MVPA at T3 remained significantly higher than at T1. There was no significant interaction effect for class group and time on in-school MVPA (Wilk’s lambda = 0.878, F (2, 20) = 1.395, *p* > 0.05, η^2^ = 0.122).

There was no significant effect for time on motivation for PA (Wilk’s lambda = 0.869, F (2, 27) = 2.308, *p* > 0.05, η^2^ = 0.146), PA self-efficacy (Wilk’s lambda = 0.934, F (2, 27) = 0.955, *p* > 0.05, η^2^ = 0.066), school affect (Wilk’s lambda = 0.990), F (1, 28) = 0.290, *p* > 0.05, η^2^ = 0.010), or peer social support (Wilk’s lambda = 0.982, F (1, 28) = 0.521, *p* > 0.05, η^2^ = 0.018). Mixed between-within ANOVA’s showed no significant interaction effect between class group and time for motivation for PA (Wilk’s lambda = 0.919, F (2, 26) = 1.138, *p* < 0.05, η^2^ = 0.081), PA self-efficacy (Wilk’s lambda = 0.999, F (2, 26) = 0.010, *p* > 0.05, η^2^ = 0.001), school affect (Pillai’s trace = 0.011, F (1, 27) = 0.290, *p* > 0.05, η^2^ = 0.011), or peer social support (Pillai’s trace = 0.008, F (1, 27) = 0.228, *p* > 0.05, η^2^ = 0.008),

A significant increase in the proportion of students displaying asset behaviour and a significant decrease in the proportion of students displaying problem behaviour from T1 to T2 was seen for School 2. It was 77% (CL ES = 0.77) more likely that more students would display asset behaviour at T2 compared to T1, and 77% (CL ES = 0.77) more likely that less students would display problem behaviours at T2 compared to T1 (Table 5).

School 3

There was a significant effect for time on in-school MVPA for School 3 (Wilk’s lambda = 0.321, F (2, 39) = 41.29, *p* < 0.000, η^2^ = 0.679). Pairwise comparisons showed a significant increase in mean in-school MVPA from T1 to T2 and mean in-school MVPA at T3 remained significantly higher than at T1. There was no significant interaction effect for class group and time on in-school MVPA (Pillai’s trace = 0.001, F (2, 38) = 0.027, *p* > 0.05, η^2^ = 0.001).

One-way repeated-measures ANOVA showed no significant change over time for motivation for PA (Wilk’s lambda = 0.998, F (2, 33) = 0.034, *p* > 0.05, η^2^ = 0.002), PA self-efficacy (Wilk’s lambda = 0.969, F (2, 32) = 0.506, *p* > 0.05, η^2^ = 0.031), school affect (Wilk’s lambda = 0.994, F (2, 32) = 0.100, *p* > 0.05, η^2^ = 0.006), or peer social support (Wilk’s lambda = 0.990, F (2, 32) = 0.154, *p* > 0.05, η^2^ = 0.010). Mixed between-within ANOVA’s showed no significant interaction effects for motivation for PA (Pillai’s trace = 0.023, F (2, 32) = 0.369, *p* > 0.05, η^2^ = 0.023), PA self-efficacy (Pillai’s trace = 0.051, F (2, 31) = 0.835, *p* > 0.05, η^2^ = 0.051), or peer social support (Pillai’s trace = 0.027, F (2, 31) = 0.437, *p* > 0.05, η^2^ = 0.027), A significant and large interaction effect for class group and time on school affect was found (Pillai’s trace = 0.185, F (2, 31) = 3.508, *p* < 0.05, η^2^ = 0.185).

In School 3, there was a significant increase in the proportion of students displaying asset behaviour and a significant decrease in the proportion of students displaying problem behaviour from T1 to T2 (Table 6). It was 88% (CL ES = 0.88) more likely that more students would display asset behaviour at T2 compared to T1, and 83% (CL ES = 0.83) more likely that less students would display problem behaviours at T2 compared to T1 (Table 6).

#### 3.3.2. Comparison across Schools

Inspection of effect sizes shows that Schools 1 and 2, who implemented 100% of the ASF success criteria (Table 2), displayed larger increases in students’ in-school MVPA over time (η^2^ = 0.84 and 0.82 respectively) compared to School 3 (η^2^ = 0.68), who implemented less than half of the ASF success criteria (Table 2). Mixed between-within ANOVA showed there was a significant and medium interaction effect between school and time (Pillai’s trace = 0.257, F (4, 208) = 7.68, *p* < 0.000, η^2^ = 0.13) for in-school MVPA. Students in each of the schools increased the number of minutes spent in MVPA during school from T1 to T2, but the increase in MVPA from T1 to T2 was greater for Schools 1 and 2 compared to School 3. In addition, students in School 2 showed a slight decrease in in-school MVPA from T2 to T3, while students in School 1 and 3 showed a slight increase in in-school MVPA. Across all three schools, the greatest change in in-school MVPA occurred between T1 and T2.

Schools 1 and 2 also displayed larger increases in students’ motivation to be physically active (η^2^ = 0.043 and 0.131 respectively), and in their PA self-efficacy (η^2^ = 0.070 and 0.066 respectively) over time compared to School 3 (η^2^ = 0.002 for motivation to be PA and η^2^ = 0.031 for PA self-efficacy). However, mixed between-within ANOVA showed no significant main or interaction effects between school and time for motivation for PA, or for PA self-efficacy.

Schools 1 and 2 displayed larger increases in school affect (η^2^ = 0.036 and η^2^ = 0.010 respectively), and in peer social support (η^2^ = 0.095 and η^2^ = 0.018) compared to School 3 (η^2^ = 0.006 for school affect, and η^2^ = 0.010 for peer social support). However, mixed between-within ANOVA showed no significant main or interaction effects between school and time for school affect or peer social support.

Inspection of effect sizes showed that School 1 had the greatest positive changes in student behaviour over time (CL ES = 0.95–0.97; Table 4), followed by School 3 (CL ES = 0.83–0.88; Table 6) and School 2 (CL ES = 0.77; Table 5). Mixed between-within ANOVA showed that for asset behaviour there was a significant and large interaction effect between school and time (Pillai’s trace = 0.172, F (2,36) = 3.74, *p* = 0.033, η^2^ = 0.17). School 1 showed the steepest increase in asset behaviour between T1 and T2, while School 2 and 3 showed a less significant increase in asset behaviour. For all schools, the proportion of students displaying asset behaviour increased from T1 to T2. For problem behaviour, there was no significant interaction effect between school and time. There was a significant and large main effect for time (Wilk’s lambda = 0.42, F (1,35) = 48.76, *p* = 0.000, η^2^ = 0.58), with the proportion of students displaying problem behaviour decreasing over time across all schools.

## 4. Discussion

Using the RE-AIM framework, key components relating to programme reach, effectiveness, adoption, implementation, and maintenance of Ireland’s ASF were evaluated in this research and are discussed in turn below.

### 4.1. Reach

As shown in Figure 1, the ASF originated from a European Union (EU) initiative, the ‘European Most Active School Award’, which commended schools who provided PA opportunities to their students. The ASF was developed with a view to expanding the reach of this EU active school initiative in a less competitive and more inclusive manner.

Since being rolled out under the auspices of the Department of Education and Skills in 2009, 85% of primary schools have engaged with the programme. Primarily, the ASF aims to reach all primary school staff and children A key method of reaching all staff is the use of a ‘guided self-evaluation’, the first step in the ASF process. The guided self-evaluation requires staff to collectively assess their school’s current PA provision. It does not form part of the ASF assessment but is the beginning of the process upon which PA promotion ideas can be developed. A significant adaptation to the ASF was the addition of the three pillars (PA, PE, and Partnerships), and the removal of co-curricular sport. Co-curricular sport, such as sports team training, was removed from the ASF success criteria requirements (by the ASF team, which is completely independent of the research team) to ensure that the ASF could reach all students equally, rather than ‘sporty’ or physically active students benefiting more from the programme than others, as has occurred in other school PA programmes which specifically include after-school sport [32]. Removing a requirement for teachers to remain after school also increased the likelihood of the ASF reaching all teachers, and thus all students. Government investment from the health and education departments, and the inclusion of the ASF in Ireland’s National Physical Activity Plan, has increased the reachability of the ASF through additional funding and support [11].

The ASF is predominantly an Irish primary school PA initiative, but recently the ASF commenced development of a second-level school version of the programme [54]. It was recognized that a different ASF model was required for the post-primary sector and a co-design approach is currently being undertaken to extend the initiative into the secondary school setting. The feasibility phase is due to end this year. Considering the significant decrease in PA in Irish adolescents following the transition to second-level school [55], and the substantially lower PA levels in Irish second-level school students compared to primary school students [8], a successful PA promotion initiative that could reduce or reverse these changes would be welcome. The successful roll out of a second-level ASF would increase the reach of the programme beyond the primary school population, and to a population that is at even greater risk of inactivity [8,55].

### 4.2. Effectiveness

The jury is currently out on whether school-based PA promotion initiatives are actually effective in doing what they primarily aim to do, that is, increase PA [20]. Where PA as an outcome is evaluated, it is often based on self-report measures [56]. Frequently, however, PA outcomes of whole-school PA promotion initiatives are not reported [56]. In light of this, a significant strength of the current research is the evaluation of the ASF’s primary goal, that is, to create physically active school communities. In considering this accelerometer MVPA data, however, it is important to acknowledge the challenges and limitations of using accelerometers, and using cut points as a measure of MVPA, as articulated in Cain et al. (2019) [49], and appraise the findings presented in that light. Preliminary results from this pilot study indicate that the ASF had a large effect on in-school MVPA, with students in-school MVPA having increased across the academic year during which the ASF was being implemented. Of particular significance is that in-school MVPA also remained higher than baseline levels at follow up. In addition, it is interesting to note that the increase in in-school MVPA was greater in the schools who fully implemented the ASF compared to the school who did not, although it must be recognised that data collected for the current study are from a single group pilot study that did not include a control group. It is recommended that whole-school PA promotion initiatives encompass multiple factors known to support positive PA behaviours [13,17]. By embracing a process-oriented approach to PA promotion and addressing multiple components within its three-pillar structure, the ASF has proven to be more effective in promoting and maintaining PA behaviour change when compared to more unidimensional outcome-oriented initiatives (for example targeting PE class, or school breaktimes alone) [57].

Just as there is a socioeconomic divide in PA participation, a socioeconomic divide in the impact of health-promotion interventions on individuals has also been reported [58], with fears that many health-promotion initiatives may exacerbate existing inequalities rather than reduce them [20]. PA outcomes from the current ASF evaluation indicate that the ASF is a successful PA promotion initiative among socioeconomically disadvantaged groups. Potential reasons for this are the inclusion of attributes within the ASF initiative that have been reported to be important for achieving successful outcomes in socioeconomically disadvantaged groups [58]. Beauchamp and colleagues found that initiatives that have a wide reach, a long duration, and which aim to alter environmental or social factors that act as barriers to PA, are more likely to achieve successful outcomes in socioeconomically disadvantaged groups, compared to initiatives that simply target individual behaviour change [58]. In the context of the ASF, it has the potential to reach all primary schools in Ireland, and requires a 1–2 year application process duration to achieve ASF status. It also includes numerous aspects within its success criteria document that aim to alter environmental and social barriers to PA, for example the provision of PE in line with curriculum guidelines, integration of PA across all curricular areas, and development of community links with local sports organisations outside of the school. Therefore, the content of the ASF includes the elements identified by Beauchamp et al. (2014) as being important when targeting behaviour change across socioeconomic groups.

As well as directly targeting children’s PA levels, successful PA promotion initiatives should consider other determinants of PA, such as motivation and self-efficacy, which are likely to result in the maintenance of PA changes over time [19]. Results from the current evaluation indicate that motivation for PA, PA self-efficacy, school affect, and peer social support did not change significantly in any of the three schools over time. In considering the reason for this, we must in part consider that immediate changes in physical activity are often motivated by extrinsic factors, for example changes made to PE class, or lunch time activity offerings. Intrinsic motivation, the form of motivation that is hypothesised to have a stronger influence on behaviour long term, however, takes time and repeated performance to develop [59]. Interventions that initially enact behaviour can alter intrinsic motivation over time, but the adopted new behaviour (e.g., physical activity) needs to resemble the children’s values and be perceived as personally relevant [60]. The lack of change in motivation in this study may also in part be explained by the fact that many tests and scales developed in psychological and educational research are considered relatively easy, and can lead high scoring participants to answer items ‘correctly’ resulting in a ceiling effect [61]. While high scores across the board in the current study may be a result of a ceiling effect, we note that this study’s cohort were from schools designated as disadvantaged, where there is a known gap reported in literacy levels [62]. Thus, while questionnaires used in this study were validated for the cohort’s age, during the data collection process the researchers identified an array of vocabulary that required constant translation (e.g., ‘benefits’, ‘expenses’, ‘value’, ‘physically active’). It is therefore possible that some participants answered ‘correctly’ without truly understanding the question they were asked. Accordingly, we propose that qualitative data collection processes, where student voices can be heard in their own words, are potentially crucial when evaluating children’s attitudes and motivations towards engagement in PA, PE and school. Qualitative results presented in a recent paper, involving a subset of participants from the current study, indicated that prior to ASF engagement the basic psychological needs associated with successful PA engagement were met for some children but certainly not all. Further, there was a reported positive shift in autonomy, relatedness and competence from baseline to follow up (one year after ASF participation) [57]

The goal of whole-school PA programmes is to effect behaviour change, and to increase PA levels in children [18,20]. While results show that in-school MVPA increased over the course of ASF implementation, it is not known whether children in this study also increased their MVPA after school. The ASF recognises the importance of community partnerships in promoting a more physically active lifestyle, thus identifying that PA opportunities happen within, and outside of, the school environment. To more comprehensively evaluate the effect of the ASF on MVPA, future evaluations should monitor whole-day PA changes during ASF implementation to identify whether the ASF programme is impacting behaviour beyond the school environment. That being said, considering children spend almost half of their day in school [63], any increases in school-day MVPA are evidently beneficial. Furthermore, qualitative findings from McGann and colleagues (2020) suggest that ASF participation not only had positive impact on children’s in-school PA levels but also positively impacted participants perceptions and motivations towards PA. These are described as key determinants of sustained engagement in PA over time [19].

### 4.3. Adoption

The ASF has been in existence for over ten years, and adoption figures in Ireland are indicative of its success thus far, with 85% of primary schools having engaged with the programme at some point over the past ten years. Recently, the ASF has been adopted outside of Ireland, with primary schools in Italy and Lithuania trialling the ASF programme with a view to addressing poor PA levels among children [64].

One element of the ASF that may have enhanced adoption within an Irish context is the use of a flag to reward, and incentivise, adoption. Flags are highly relevant in an Irish primary school context, and are currently used in other behaviour change programmes in schools such as the Green Flag for Environmental Awareness (www.greenschoolsireland.org), and the Yellow Flag for Inclusion and Diversity (www.yellowflag.ie). Awarding a flag as part of the ASF process makes a statement about the work a school has put in to achieve success in PA promotion, and has been previously identified as a motivating factor for adoption among teachers [21,27].

Another important factor which has enhanced adoption of the ASF by primary schools is buy in from multiple stakeholders [14,18]. The ASF was initially run solely under the auspices of the Department of Education, the ASF core funder. In 2015, in addition to the Department of Education, the ASF received sponsorship from the Department of Health, under Healthy Ireland. Since then, the ASF has been jointly funded by both government departments. This serves as an endorsement at government level of the ASF programme. Additional funding gained through the involvement of Healthy Ireland with the ASF has also provided an opportunity to incentivise adoption of the ASF through the provision of PA resources such as playground leader vests, badges, whistles, walkway packs. These resources are only provided at ASF training workshops, thereby incentivising attendance and thus increasing the likelihood of implementation fidelity through education [18,22].

Another government-level endorsement of the ASF, which promotes adoption of the programme, is the inclusion of the ASF within the National Physical Activity Plan for Ireland [11]. Healthy Ireland, under the Department of Health, launched Ireland’s first ever National PA Plan in 2016, and in this committed to extending the ASF by 500 additional schools by 2020. The most recently published review of the National PA Plan was published in 2018, and indicated that, at that time, there was a net increase of 409 schools holding current ASF status since the Plan was published in 2016 [65].

Despite the positive adoption rates of the ASF within Ireland’s primary schools so far, not all schools are inclined to adopt this programme. The representativeness of schools engaging with the ASF is key to understanding how best to target schools with low adoption rates. A larger proportion of non-DEIS (32%) schools are engaged with the ASF, compared to DEIS schools (21%). Paperwork, time commitments [21,28], and specific barriers for DEIS schools such as low literacy levels of pupils and parents [21], have been cited by teachers as barriers to adoption of the ASF. Specifically, surveying parents and students, required as part of the ASF, has been identified by DEIS-school teachers as a significant challenge for implementation of the ASF [21]. McGann et al. (2020) suggests allowing schools to adapt survey instruments to suit the literacy levels of students and parents within the school. Indeed, ASF programme developers present as being cognisant of potential socioeconomic and school-level barriers to adoption, and list one of the strengths of the ASF as ‘taking on board feedback from school [and using] research results, to improve adoption going forward’.

### 4.4. Implementation

A recent meta-analysis evaluating the effectiveness of whole-school PA programmes in achieving increases in PA found no evidence that these programmes are successful [20]. Implementation infidelity was highlighted as one of the main problems in evaluating such initiatives [20]. If evaluators cannot ascertain the fidelity with which a programme is implemented, then it is impossible to say that the programme does or does not work. The ASF utilise success criteria as ‘targets’ which allow schools to track their progress over time. Success criteria allow schools to respond to the needs of the children in a way that best suits their circumstances. While there is flexibility in terms of the timing and order of success criteria delivery, ultimately all targets must be met in order to prove implementation fidelity. The ASF’s use of ‘success criteria’, which is the basis by which ASF status is awarded or not, could prove useful to other whole-school PA programmes. The success criteria were introduced into the ASF process following feedback from teachers which highlighted the need to address time constraints previously associated with implementing the ASF.

Investment and support from key stakeholders help to equip PA promotion programmes with the training resources to be successful in terms of implementation fidelity. Access to programme support and education for teachers is an essential element of successful whole-school PA promotion initiatives [18]. Indeed, Daly-Smith et al. (2020) suggest that resources for PA promotion should be made available for sharing between programme settings and agents [14]. Within the ASF programme, there are three workshops for teachers (‘Find Out More’, ‘Getting Started’, ‘Nearly There’) over the course of a school year [14]. This has evolved from just a single workshop through an increase in funding when Healthy Ireland came on board in 2015. Workshops not only afford opportunities for coordinators to engage in a community of practice and the sharing of intellectual ideas, but also act as an opportunity for the ASF to disseminate physical resources. To that end, the ASF provide schools with a number of sought after resources that attract coordinators to meetings and, support implementation fidelity. Resources include high quality playground leader ‘bibs’ and whistles (associated with the PA pillar), metal plaques and wall signs to support schools in the setup of a personalised ‘Walkway’ route (associated with the PA pillar) and, committee badges (associated with the partnerships pillar).

The strict and comprehensive nature of the success criteria ensure implementation fidelity, and that every child in an ASF school is experiencing the full benefits of the programme as intended by the ASF. The workload for teachers, however, is a barrier to implementation of the programme [21,28]. In the context of the current evaluation, for School 3, the PA pillar success criteria were the primary criteria implemented. Further qualitative research may be needed to explore the reasons for this focus. That being said, despite having only achieved under half of the total success criteria, an increase in in-school MVPA was still found among children in School 3. Compromising on the success criteria is not an option, nor should it be, as evidently the ASF is a successful intervention in terms of MVPA outcomes. However, it may be that incremental awards, or ‘mini-flags’ throughout the course of the ASF could encourage schools to stay the course and incentivise the programme for schools.

### 4.5. Maintenance

The inclusion of the ASF in Ireland’s National Physical Activity Plan [11], funded by Healthy Ireland, is indicative of this initiative being adopted, and maintained, at a national level. Continued funding support from government departments (Department of Education and Skills, and Department of Health) also suggests support for the ASF within Irish education and health structures.

Over the course of the ASF’s 10 year existence, the unique focus on ‘renewal’ which comes with an explicit ‘renewal process’ has grown. Renewal schools are those which have had the Flag for a three-year period, and choose to renew their status as an Active School (www.activeschoolflag.ie) by re-engaging with the process, confirming that they are still meeting all of the ASF criteria and completing an additional set of success renewal criteria. An analysis of interviews with coordinators from renewal schools by McGann and colleagues (2020) found that teachers felt the renewal process offered an opportunity to rejuvenate success criteria and to ensure that they ‘kept the system going’ [21]. Coordinators were identified as being motivated to renew the ASF owing to (i) a litany of perceived benefits associated with ASF implementation in their school and (ii) a desire to not let the hard work previously put in by the school team (to attain the initial flag) go to waste. Current figures show that 34% of schools presently engaged in the ASF are renewal schools. This indicates that there is a relatively strong appetite in schools to continue engagement with the ASF after initial involvement. That being said, out of the 2635 schools who registered with the ASF, 38% (1013 schools) did not go on to receive a flag. (Figure 3). In addition, we estimate that the renewal rate (those schools attaining the flag for a 2nd time) is approximate 39%. The reason for drop out, or disengagement, should be further explored to better understand how to increase maintenance of the initiative in primary schools. The importance of maintaining schools in the ASF is twofold. Firstly, it means that schools adopt and maintain the behaviour changes that the ASF desires. Secondly, it allows for the sharing of knowledge and resources from renewal schools to new schools, which is acknowledged as being key to the successful implementation and maintenance of whole-school PA promotion programmes [14].

## 5. Conclusions

In order for an in-school PA intervention to be effective, it needs to ‘work’ for both the teachers and children alike. From the perspective of the teachers, one of the real strengths of the ASF is their apparent ability to collect, process and utilise feedback from teachers, coordinators and research in order to continuously adapt the process in line with participant needs. This adaption has been evident over the last ten years. One of the most important adaptions presents as the introduction of success criteria which facilitate implementation fidelity within schools, while the introduction of the renewal process encourages and supports maintenance at a school-level.

From the perspective of the child, if we take an increase in PA levels as a basic requirement for success in a PA intervention initiative, evidence from the current study suggests that the ASF is an effective PA promotion initiative in primary schools in the short-to-medium term. PA measurement before, during, and after programme implementation, showed a significant increase in students’ in-school MVPA, albeit in a single group pilot study. Importantly, this evaluation showed the ASF to be an effective PA promotion initiative in socioeconomically disadvantaged schools, where health-promotion programmes are often found to be unsuccessful. Whether such successful in-school PA promotion programmes can be effective not just in-school, but in improving PA behaviours outside of the school environment, is an area which is worth exploring in the future.

The ASF’s success, not just in terms of its effectiveness, but in its reach, adoption, implementation and maintenance, can serve as a guide to inform future similar PA promotion programmes. Future research considering a cost–benefit analysis of the ASF, including some of the information generated in the current study, but building on this with information from a broader sample of schools, may prove valuable to further supporting sustained rollout of the programme.

## Figures and Tables

**Table 1:** Definitions and data sources for each of the RE-AIM dimensions.

RE-AIM Dimension	Definition	Level of Analysis	Data Sources
Reach	Factors within the ASF structure that enhance programme reach	(1) Administration	ASF National Coordinator interview
Effectiveness	Effect of the ASF on target outcome measures (e.g., MVPA, classroom behaviour, motivation for PA, PA self-efficacy, school affect, peer and social support)	(3) Outcomes	Accelerometer-measured student in-school MVPA Teacher questionnaire Student questionnaire
Adoption	Number and representativeness of primary schools adopting the ASF	(1) Administration	ASF Administration Office records
Implementation	Factors within the ASF structure that promote implementation fidelity	(1) Administration	ASF National Coordinator interview
	Level of implementation fidelity adhered to by schools	(2) Application	School ASF success criteria document
Maintenance	Factors within the ASF structure that indicate and support programme maintenance over time	(1) Administration	ASF National Coordinator interview ASF Administration Office records ASF website

Note: ASF = Active School Flag; MVPA = moderate–vigorous physical activity.

**Table 2:** ASF success criteria achieved by each case study school.

ASF Pillar	Success Criteria	S1	S2	S3
Physical Education	1. As a whole staff we completed the ASF self-evaluation document for PHYSICAL EDUCATION and submitted it at the start of the process.	✓	✓	✓
	2. Our school website/PowerPoint includes a section about PHYSICAL EDUCATION.	✓	✓	✗
	3. All pupils are provided with 60 min timetabled PE per week, as a minimum.	✓	✓	✓
	4. All classes are taught at least 5 different PE strands each year (Athletics, Aquatics, Dance, Gymnastics, Games, Outdoor and Adventure).	✓	✓	✓
	5. Our school PE programme allocates a balanced amount of teaching time to each of the different PE strands.	✓	✓	✗
	6. Our school prioritises a different PE strand for further development every year.	✓	✓	✗
	7. All teachers use the PSSI lesson plans to guide their delivery of the Primary PE Curriculum.	✓	✓	✓
	8. Our school ensures that all PE activities are planned so that they are accessible by all pupils.	✓	✓	✗
	9. Every child’s progress in PE is discussed with parents at PT meetings and feedback is included in the annual school report.	✓	✓	✗
	10. Members of staff have undertaken CPD in PE during the past 12 months. New knowledge, ideas and resources have been shared out amongst staff members.	✓	✓	✗
	11. Our school carries out a PE equipment audit once a year, disposing of old and broken equipment.	✓	✓	✓
	12. Our PE equipment and resources are clearly labelled, well organised and easily accessible.	✓	✓	✓
Physical Activity	1. As a whole staff we completed the ASF self-evaluation document for PHYSICAL ACTIVITY and submitted it at the start of the process.	✓	✓	✓
	2. Our school website/PowerPoint includes a section about PHYSICAL ACTIVITY.	✓	✓	✗
	3. Our school provides twice daily playground breaks for all classes.	✓	✓	✓
	4. Children are allowed to run during playground breaks.	✓	✓	✓
	5. Our school yard is zoned to allow children to engage in a variety of different activities.	✓	✓	✓
	6. Our school facilitates the use of sports equipment during break times.	✓	✓	✓
	7. Our school trains pupils as Playground Leaders.	✓	✓	✓
	8. Our school has sign posted an ‘Active School WALKWAY’.	✓	✓	✗
	9. Children and staff are encouraged ‘Do Your Talking as You’re Walking’ during breaktimes.	✓	✓	✗
	10. Every class incorporates short Physical Activity breaks into their daily routine.	✓	✓	✓
	11. It is school policy that on the days when children are unable to play outdoors, that every classroom teacher will incorporate an e✗tended classroom-based physical activity break into the school day.	✓	✓	✗
	12. All classes participated in a non-competitive RUNNING initiative this year that lasted 4 weeks, or longer.	✓	✓	✓
	13. Our school incorporates physical activity into end of term days throughout the school year.	✓	✓	✗
	14. Our school ensures that all ASF activities are planned so that they are accessible by all pupils.	✓	✓	✗
Partnerships	1. As a whole staff we completed the ASF self-evaluation document for PARTNERSHIPS and submitted it at the start of the process.	✓	✓	✓
	2. Our school website/PowerPoint includes a section about PARTNERSHIPS.	✓	✓	✗
	3. Our school established an ASF committee at the outset of the process and pupil members were given leadership roles.	✓	✓	✓
	4. Senior pupils conducted a ‘What Club Are You In?’ survey to find out about physical activity opportunities in the local community.	✓	✓	✗
	5. Our school acknowledges physical activity and sports achievements achieved during and outside of school hours.	✓	✓	✓
	6. Our school informs pupils and parents about physical activity opportunities that are available in the local community.	✓	✓	✗
	7. Our school has emailed the Local Sports Partnership to let them know that we are working towards/renewing the ASF.	✓	✓	✗
	8. Our school has emailed the HSE Health Promotion Officer for the area to let them know that we are working towards/renewing the ASF.	✓	✓	✗
	9. Our school has sought advice and support to ensure that children with special needs can avail of all PE and physical activity opportunities.	✓	✓	✓
Active School Week	1. As a whole staff we completed the ASF self-evaluation document for ACTIVE SCHOOL WEEK and submitted it at the start of the process.	✓	✓	✗
	2. Our school website/PowerPoint includes a section about ACTIVE SCHOOL WEEK.	✓	✓	✗
	3. Our school commits to having ‘Active School Week’ (ASW) as part of the annual school calendar.	✓	✓	✗
	4. Our school ensures that all ASW activities are planned so that they are accessible by all pupils.	✓	✓	✗
	5. Our school involves pupils in the design and organisation of the ASW programme.	✓	✓	✗
	6. Tracksuits replaced uniforms for staff and pupils for the duration of ASW.	✓	✓	✗
	7. Our school promoted physical activity in a cross-curricular way throughout ASW.	✓	✓	✗
	8. Our school gives physical activity tasks as homework during ASW.	✓	✓	✗
	9. Our school organised a whole-school FUN event during ASW.	✓	✓	✗
	10. Out school invites parents to participate in ASW activities.	✓	✓	✗
	11. Our school invited local sports clubs and physical activity providers to give taster sessions during ASW.	✓	✓	✗
	12. Our school made use of local physical activity amenities during ASW (playgrounds, forest trails, etc).	✓	✓	✓
	ACTIVE SCHOOL FLAG AWARDED	✓	✓	✗

Note: S1—School 1, S2—School 2, and S3—School 3; S1 and S2 achieved ASF status, but S3 did not achieve ASF status.

**Table 3:** Descriptive statistics for participants in each case study school.

	N	Age (Years)	Female (%)	Male (%)
School 1				
3rd Class	21	9.05 ± 0.92	48	52
5th Class	29	10.83 ± 0.66	55	45
School 2				
3rd Class	18	8.72 ± 0.67	33	67
5th Class	12	10.75 ± 0.45	25	75
School 3				
3rd Class	18	8.94 ± 0.64	50	50
5th Class	28	11.00 ± 0.67	54	46

**Table 4:** School 1—mean scores and change over time for outcome variables.

School 1							
	N	T1	T2	T3	η^2^	90% CI	
MVPA (mins)	43	17.50 ± 7.55	28.06 ± 8.53	29.33 ± 8.66	0.842	0.751–0.878	
School Affect	40	4.29 ± 0.734	4.43 ± 0.677	4.33 ± 0.603	0.036	0.000–0.135	
Peer Social Support	40	4.26 ± 0.590	4.01 ± 0.750	4.01 ± 0.750	0.095	0.0001–0.250	
Motivation for PA	36	45.11 ± 6.29	44.28 ± 8.20	46.19 ± 7.30	0.043	0.000–0.154	
PA Self-Efficacy	40	20.60 ± 4.61	20.73 ± 2.15	20.83 ± 2.04	0.070	0.000–0.193	
	**N**	**T1**	**T2**	***t***	***p***	**Hedges *g_av_* [95%CI]**	**CL ES**
Asset Behaviour	13	4.56 ± 0.41	5.38 ± 0.57	6.97	0	1.54 [0.54–1.08]	0.97
Problem Behaviour	13	1.74 ± 0.58	1.12 ± 0.48	5.91	0	1.09 [0.39–0.85]	0.95

Note: MVPA = moderate–vigorous physical activity (3rd and 5th class students only); η^2^ = eta squared; CI = confidence interval; CL ES = common language effect size.

**Table 5:** School 2—mean scores and change over time for outcome variables.

School 2							
	N	T1	T2	T3	η^2^	90% CI	
MVPA (mins)	23	20.23 ± 5.64	34.36 ± 10.81	33.33 ± 8.90	0.817	0.644–0.867	
School Affect	29	4.28 ± 0.671	4.34 ± 0.663	4.34 ± 0.663	0.010	0.000–0.133	
Peer Social Support	29	4.08 ± 0.627	3.96 ± 0.782	3.96 ± 0.782	0.018	0.000–0.156	
Motivation for PA	29	42.83 ± 7.31	42.72 ± 6.47	44.97 ± 6.77	0.146	0.000–0.309	
PA Self-Efficacy	29	20.83 ± 2.51	21.34 ± 2.16	21.41 ± 2.23	0.066	0.000–0.205	
	**N**	**T1**	**T2**	***t***	***p***	**Hedges *g* [95% CI]**	**CL ES**
Asset Behaviour	11	5.00 ± 1.17	5.43 ± 0.63	2.41	0.04	0.42 [0.03–0.83]	0.77
Problem Behaviour	10	1.80 ± 0.94	1.52 ± 0.67	2.3	0.05	0.31 [0.004–0.56]	0.77

Note: MVPA = moderate–vigorous physical activity; η^2^ = eta squared; CI = confidence interval; CL ES = common language effect size.

**Table 6:** School 3—mean scores and change over time for outcome variables.

School 3							
	N	T1	T2	T3	η^2^	90% CI	
MVPA (mins)	41	18.78 ± 8.50	24.94 ± 8.11	25.36 ± 8.08	0.679	0.511–0.754	
School Affect	34	4.28 ± 1.09	4.35 ± 0.77	4.32 ± 0.78	0.006	0.000–0.062	
Peer Social Support	34	4.02 ± 0.854	3.95 ± 0.800	3.92 ± 0.785	0.010	0.000–0.076	
Motivation for PA	35	41.91 ± 9.89	42.03 ± 9.66	41.63 ± 10.34	0.002	0.000–0.038	
PA Self-Efficacy	34	20.97 ± 2.71	20.38 ± 2.76	20.47 ± 2.69	0.031	0.000–0.132	
	**N**	**T1**	**T2**	***t***	***p***	**Hedges *g* [95% CI]**	**CL ES**
Asset Behaviour	15	4.58 ± 0.84	4.98 ± 0.71	4.62	0.000	0.49 [0.21–0.59]	0.88
Problem Behaviour	15	2.30 ± 0.85	1.96 ± 0.59	3.62	0.000	0.45 [0.14–0.54]	0.83

Note: MVPA = moderate–vigorous physical activity; η^2^ = eta squared; 95% CI = 95% confidence interval; CL ES = common language effect size.
